# Supplementary material for: A convolutional neural network highlights mutations relevant to antimicrobial resistance in Mycobacterium tuberculosis
Source: Nat Commun. 2022 Jul 2;13:3817. doi: 10.1038/s41467-022-31236-0 (PMC9250494; doi:10.1038/s41467-022-31236-0)

Supplementary Table 1: Comparison of models by predictive performance. Mean AUCs for first-line drugs and mean AUCs for second-line drugs are compared between logistic regression + L2 regularization and the MD-CNN (LR v MD-CNN), logistic regression + L2 regularization and the WDNN (LR v WDNN), and so on. Welch's two-sample, two-sided t-test is used to determine the statistical significance of the difference in mean AUCs between models. Benjamini-Hochberg p-value correction is performed for multiple testing. Note that ethionamide, and levofloxacin are excluded from the list of second-line drugs because the original WDNN was not trained on these drugs. LR = logistic regression + L2 regularization; WDNN = wide and deep neural network; MD-CNN = multi-drug convolutional neural network; SD-CNN = single-drug convolutional neural network.

	**Drug group**	**Model 1**	**Model 1 AUC**	**Model 2**	**Model 2 AUC**	**Difference in mean AUCs [95% CI]**	**p**	**Benjamini-Hochberg p**
**Model comparison**								
**LR v MD-CNN**	First-line drugs	LR	0.923	MD-CNN	0.948	-0.026 [-0.047, -0.0039]	0.032	0.059
**LR v WDNN**	First-line drugs	LR	0.923	WDNN	0.960	-0.037 [-0.058, -0.016]	0.0022	0.0054
**LR v SD-CNN**	First-line drugs	LR	0.923	SD-CNN	0.938	-0.015 [-0.037, 0.006]	0.18	0.18
**WDNN v MD-CNN**	First-line drugs	WDNN	0.960	MD-CNN	0.948	0.011 [-0.0015, 0.024]	0.10	0.13
**WDNN v SD-CNN**	First-line drugs	WDNN	0.960	SD-CNN	0.938	0.022 [0.0096, 0.034]	0.0014	0.0042
**MD-CNN v SD-CNN**	First-line drugs	MD-CNN	0.948	SD-CNN	0.938	0.010 [-0.0034, 0.024]	0.16	0.18
**LR v MD-CNN**	Second-line drugs	LR	0.860	MD-CNN	0.912	-0.051 [-0.081, -0.022]	0.0010	0.0042
**LR v WDNN**	Second-line drugs	LR	0.860	WDNN	0.924	-0.064 [-0.092, -0.036]	0.000044	0.00053
**LR v SD-CNN**	Second-line drugs	LR	0.860	SD-CNN	0.888	-0.028 [-0.058, 0.0028]	0.082	0.12
**WDNN v MD-CNN**	Second-line drugs	WDNN	0.924	MD-CNN	0.912	0.013 [-0.0055, 0.031]	0.18	0.18
**WDNN v SD-CNN**	Second-line drugs	WDNN	0.924	SD-CNN	0.888	0.036 [0.016, 0.057]	0.00083	0.0042
**MD-CNN v SD-CNN**	Second-line drugs	MD-CNN	0.912	SD-CNN	0.888	0.024 [0.0023, 0.045]	0.034	0.059

Supplementary Table 2: Comparison of models by predictive performance, measured as mean sensitivity and mean specificity for logistic regression + L2 regularization, WDNN (wide and deep neural network), MD-CNN (multi-drug convolutional neural network), and SD-CNN (single-drug convolutional neural network). For each drug, highest sensitivity and specificity values are emboldened. The WDNN was not initially trained on levofloxacin or ethionamide and thus it is not evaluated for these drugs.

	**Logistic Regression**		**WDNN**		**MD-CNN**		**SD-CNN**	
**Drug**	**Sensitivity, %**	**Specificity, %**	**Sensitivity, %**	**Specificity, %**	**Sensitivity, %**	**Specificity, %**	**Sensitivity, %**	**Specificity, %**
Rifampicin	90.9	95.5	92.6	96.2	**96.9**	94.4	93.1	**97.2**
Isoniazid	90.6	95.7	92.0	97.0	**96.4**	93.6	91.8	**97.0**
Pyrazinamide	79.0	87.7	87.8	90.5	**88.5**	89.3	87.6	**95.1**
Ethambutol	85.1	84.4	**90.6**	85.5	86.1	**91.9**	87.5	87.3
Streptomycin	85.1	85.7	89.0	85.5	**89.7**	87.4	88.3	**89.2**
Levofloxacin	87.2	84.0	-	-	**97.4**	90.9	90.9	**94.2**
Capreomycin	72.3	88.4	78.1	88.1	**89.1**	80.3	70.8	**95.1**
Amikacin	68.6	96.1	74.1	95.6	**93.4**	82.1	72.4	**96.4**
Moxifloxacin	71.7	84.0	71.8	90.2	**87.9**	78.9	67.8	**95.0**
Ofloxacin	68.6	94.3	78.3	93.7	**92.3**	84.8	77.5	**96.4**
Kanamycin	77.2	94.9	83.9	95.5	**93.9**	88.3	84.4	**97.2**
Ethionamide	70.8	77.6	-	-	**77.2**	**81.7**	74.1	77.7
Ciprofloxacin	97.5	91.0	98.3	97.1	**99.2**	**98.3**	94.9	98.2

Supplementary Table 3: Comparison of the MD-CNN model with the WHO catalog, using mean sensitivity and mean specificity for the MD-CNN (multi-drug convolutional neural network), and the WHO (World Health Organization) catalog for each drug. The WHO catalog has no information on ofloxacin and ciprofloxacin, hence their exclusion.

	MD-CNN		WHO catalog	
**Drug**	**Sensitivity, %**	**Specificity, %**	**Sensitivity, %**	**Specificity, %**
Rifampicin	96.9	94.4	93.2	96.2
Isoniazid	96.4	93.6	87.8	97.4
Pyrazinamide	88.5	89.3	61.7	96.5
Ethambutol	86.1	91.9	78.8	89.1
Streptomycin	89.7	87.4	64.9	95.6
Levofloxacin	97.4	90.9	92.3	94.2
Capreomycin	89.1	80.3	67.8	96.0
Amikacin	93.4	82.1	71.2	97.5
Moxifloxacin	87.9	78.9	66.2	95.1
Kanamycin	93.9	88.3	83.5	97.2
Ethionamide	77.2	81.7	65.5	79.5

Supplementary Table 4: CNN models generalize on new data with realistic resistance proportions as measured by sensitivity and specificity of the CNN models' resistance predictions based on genomic data from the CRyPTIC database (see **Methods**) not otherwise used during their training and cross-validation. Sensitivity and specificity are calculated for both complete data and data down-sampled to limit the proportion of rifampicin-resistant tuberculosis strains to 5%, their estimated overall global prevalence. Down-sampling is performed 10 times, for which mean resistance numbers and proportions are calculated. The drugs below represent those antibiotics for which phenotype information is available in the CRyPTIC database. MD-CNN = multi-drug convolutional neural network; SD-CNN = single-drug convolutional neural network; 95% CI = 95% confidence interval.

	**MD-CNN complete data**				**MD-CNN down-sampled data**			
**Drug**	**Resistant (n)**	**Proportion**	**Sensitivity, % [95% CI]**	**Specificity, % [95% CI]**	**Resistant (mean n)**	**Proportion (mean)**	**Sensitivity, % [95% CI]**	**Specificity, % [95% CI]**
Rifampicin	2791	0.402	97.6 [97.6-97.7]	95.3 [95.3-95.3]	219	0.062	97.7 [97.5-97.9]	97.7 [97.5-97.9]
Isoniazid	3556	0.489	96.0 [95.9-96.0]	94.5 [94.5-94.6]	191.5	0.052	97.8 [97.6-98.0]	97.0 [96.8-97.2]
Ethambutol	1170	0.234	95.6 [95.6-95.7]	88.3 [88.2-88.4]	83.8	0.030	97.2 [96.9-97.5]	97.6 [97.3-97.9]
Levofloxacin	973	0.159	82.1 [82.0-82.3]	97.8 [97.7-97.9]	64.2	0.020	84.8 [84.0-85.7]	99.0 [98.1-99.8]
Amikacin	431	0.607	85.4 [85.2-85.6]	90.3 [90.1-90.5]	29.5	0.008	88.7 [87.7-89.8]	98.3 [97.2-99.3]
Moxifloxacin	794	0.147	91.4 [91.3-91.5]	92.4 [92.3-92.6]	55.0	0.020	95.1 [94.5-95.6]	98.6 [98.0-99.2]
Kanamycin	550	0.076	80.0 [79.9-80.2]	91.2 [91.1-91.4]	36.9	0.010	83.6 [82.5-84.8]	98.5 [97.3-99.6]
Ethionamide	929	0.147	77.5 [77.3-77.6]	91.4 [91.3-91.6]	53.9	0.015	80.9 [79.8-82.0]	98.1 [97.0-99.2]

	**SD-CNN complete data**				**SD-CNN down-sampled data**			
**Drug**	**Resistant (n)**	**Proportion**	**Sensitivity, % [95% CI]**	**Specificity, % [95% CI]**	**Resistant (mean n)**	**Proportion (mean)**	**Sensitivity, % [95% CI]**	**Specificity, % [95% CI]**
Rifampicin	2791	0.402	96.6 [96.6-96.6]	97.1 [97.1-97.2]	219	0.062	96.8 [96.5-97.0]	98.6 [98.4-98.8]
Isoniazid	3556	0.489	94.8 [94.7-94.8]	98.0 [98.0-98.1]	191.5	0.052	96.0 [95.7-96.2]	98.5 [98.2-98.8]
Ethambutol	1170	0.234	94.4 [94.4-94.5]	89.5 [89.4-89.5]	83.0	0.029	95.9 [95.5-96.3]	95.5 [95.1-95.9]
Levofloxacin	973	0.159	89.1 [89.0-89.2]	97.9 [97.8-98.0]	64.6	0.020	92.0 [91.4-92.6]	99.3 [98.7-99.9]
Amikacin	431	0.607	85.8 [85.6-85.9]	97.3 [97.1-97.4]	29.6	0.008	89.3 [88.3-90.3]	98.8 [97.9-99.8]
Moxifloxacin	794	0.147	93.6 [93.6-93.7]	94.9 [94.8-95.0]	54.9	0.020	96.3 [95.9-96.8]	98.8 [98.3-99.3]
Kanamycin	550	0.076	84.7 [84.5-84.8]	97.7 [97.6-97.9]	37.2	0.010	89.0 [88.0-89.9]	99.5 [98.5-100.0]
Ethionamide	929	0.147	73.7 [73.6-73.8]	96.8 [96.6-96.9]	52.7	0.015	71.7 [70.6-72.8]	98.1 [97.0-99.2]

Supplementary Table 5: High saliency sites according to DeepLIFT reflect a high proportion of known resistance-conferring variants. Percent of high saliency sites for each SD-CNN (single-drug convolutional neural network) model that are classified as known to be involved in antibiotic resistance.

drug	*percent known, top 1%*	*percent known, top 0.1%*
AMIKACIN	11.5%	75.0%
CAPREOMYCIN	16.9%	57.1%
ETHAMBUTOL	8.3%	61.5%
ISONIAZID	16.1%	44.4%
KANAMYCIN	14.9%	87.5%
LEVOFLOXACIN	6.3%	75.0%
MOXIFLOXACIN	12.5%	100.0%
OFLOXACIN	10.4%	100.0%
PYRAZINAMIDE	85.4%	90.0%
RIFAMPICIN	57.0%	100.0%
STREPTOMYCIN	36.4%	85.7%

Supplementary Table 6: Newly discovered sites associated with antibiotic resistance. Sites in the top 0.1% of saliency scores for any drug that are not designated as known to cause resistance in the WHO catalog.

position	locus	gene	Max saliency score	drug
7585	gyrBA	gyrA	0.44793767	LEVOFLOXACIN
1472337	rrs-rrl	rrs	0.33726892	AMIKACIN
1472358	rrs-rrl	rrs	4.3074465	STREPTOMYCIN
1472358	rrs-rrl	rrs	1.2354082	CAPREOMYCIN
1474001	rrs-rrl	rrl	1.4282935	CAPREOMYCIN
1474001	rrs-rrl	rrl	0.18678115	AMIKACIN
1476056	rrs-rrl	rrl	0.75932384	CAPREOMYCIN
1673423	FabG1-inhA	intergenic	4.7082443	ISONIAZID
2288944	pncA	pncA	6.616614	PYRAZINAMIDE
2715338	eis	intergenic	2.4716604	KANAMYCIN
2726139	oxyR-ahpC	intergenic	5.018302	ISONIAZID
2726141	oxyR-ahpC	intergenic	4.3265367	ISONIAZID
2726142	oxyR-ahpC	intergenic	4.2414837	ISONIAZID
2726145	oxyR-ahpC	intergenic	4.2477694	ISONIAZID
4243217	embCAB	intergenic	2.8458147	ETHAMBUTOL
4243222	embCAB	intergenic	3.7729878	ETHAMBUTOL
4243225	embCAB	intergenic	2.9434128	ETHAMBUTOL
4247402	embCAB	embB	2.7454894	ETHAMBUTOL
4249518	embCAB	embB	2.9824004	ETHAMBUTOL
4327484	ethAR	intergenic	2.4312048	ETHIONAMIDE

Supplementary Table 7: Lineage-defining variants represent a small fraction of high saliency positions in the SD-CNN (single-drug convolutional neural network) models. The number of sites per SD-CNN model, the number of those sites that have been mutated (SNP or INDEL) at least once, the number of sites that are correlated with lineage-defining variants **(Methods)**, the number of hits (sites that comprise the top 0.1% of high-saliency positions), and the number of hit sites that are correlated with lineage are shown.

drug	*N sites*	*N sites mutated*	*N lineage sites*	*N hits*	*N lineage hits*	*% lineage sites*	*% lineage hits*
AMIKACIN	12168	3997	3	121	2	0.08%	1.65%
CAPREOMYCIN	12168	3384	2	121	1	0.06%	0.83%
ETHAMBUTOL	20582	3468	19	205	11	0.55%	5.37%
ISONIAZID	14940	6161	15	149	5	0.24%	3.36%
KANAMYCIN	12168	3997	3	121	1	0.08%	0.83%
LEVOFLOXACIN	4859	1410	8	48	1	0.57%	2.08%
MOXIFLOXACIN	4859	1410	8	48	3	0.57%	6.25%
OFLOXACIN	4859	1410	8	48	4	0.57%	8.33%
PYRAZINAMIDE	17864	4820	10	178	4	0.21%	2.25%
RIFAMPICIN	7910	1955	9	79	4	0.46%	5.06%
STREPTOMYCIN	18252	4258	3	182	3	0.07%	1.65%

Supplementary Figure 1: Comparison of model architectures for MD-CNN. Data are presented as AUCs from five-fold cross-validation for each drug, with results for each of the three multi-drug convolutional neural network architectures. We perform five-fold cross validation using our training data (N=10,201 isolates) for three architectures: a 2x conv-conv-pool architecture with filter size 12, a 2x conv-pool architecture with filter size 12, and 2x conv-pool architecture with filter size 21. The 2x conv-conv-pool architecture was chosen for further study.

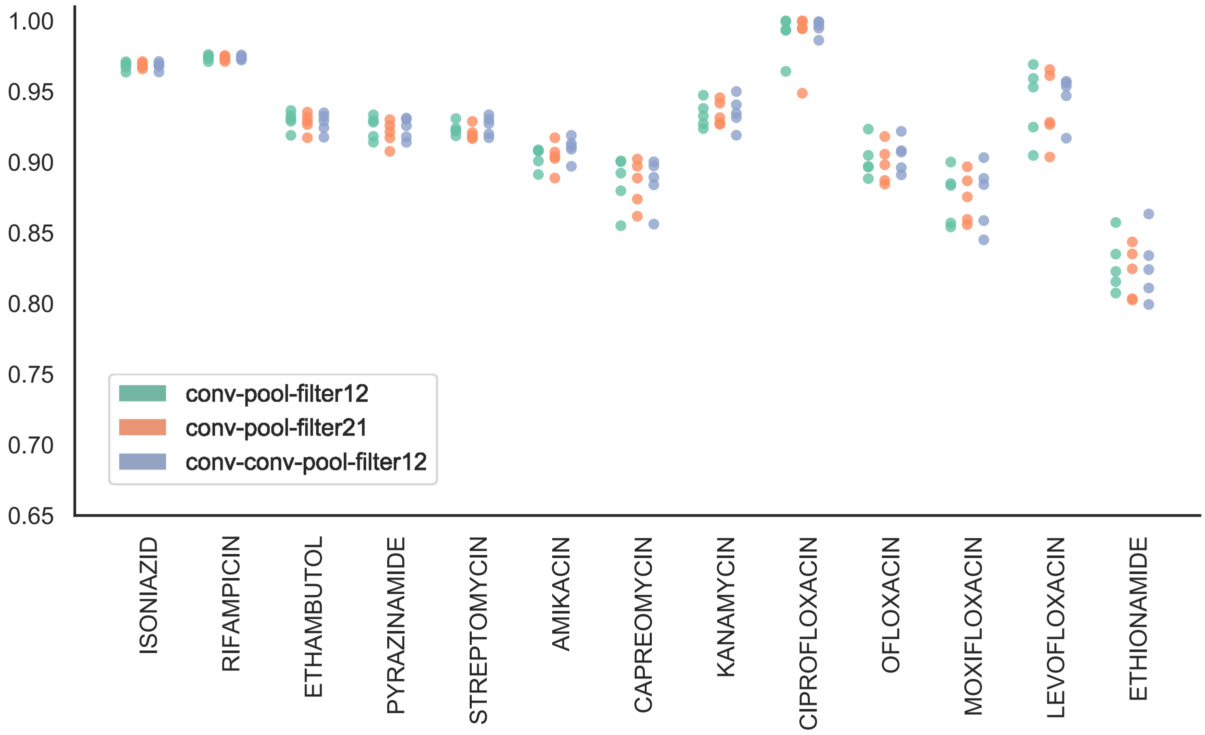

Supplementary Figure 2: schematic diagram of the single-drug convolutional neural network (SD-CNN). In the output layer, there is one node to compute a probability of resistance for the respective anti-TB drug. The input consists of '10,201' isolates (TB strains) for which there is resistance phenotype data for at least 2 anti-TB drugs; 4 for one-hot encoding of each nucleotide; no. of nucleotides in the locus/loci of interest for the respective drug (selected as detailed in **Methods**).

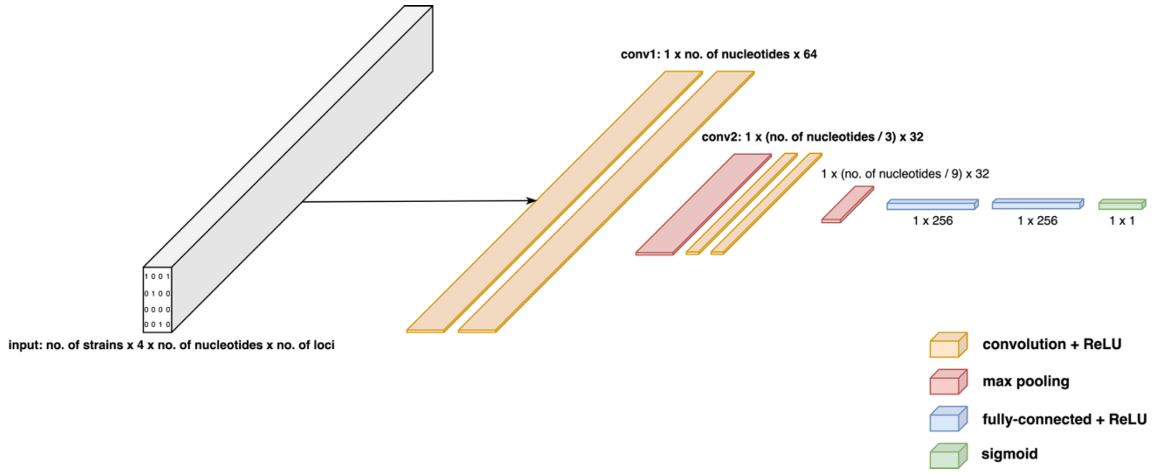

Supplementary Figure 3: Comparison of false negatives with training data phenotypes for SD-CNN. Proportion of false negatives that are nearest to resistant or sensitive isolates in the training dataset. For each falsely predicted isolate, we calculate the consensus phenotype (majority R or majority S) for the nearest isolate(s) in the training dataset. We differentiate between identical isolates (isolates with 0 mutational distance) and nearest non-identical isolates. R = resistant; S = sensitive.

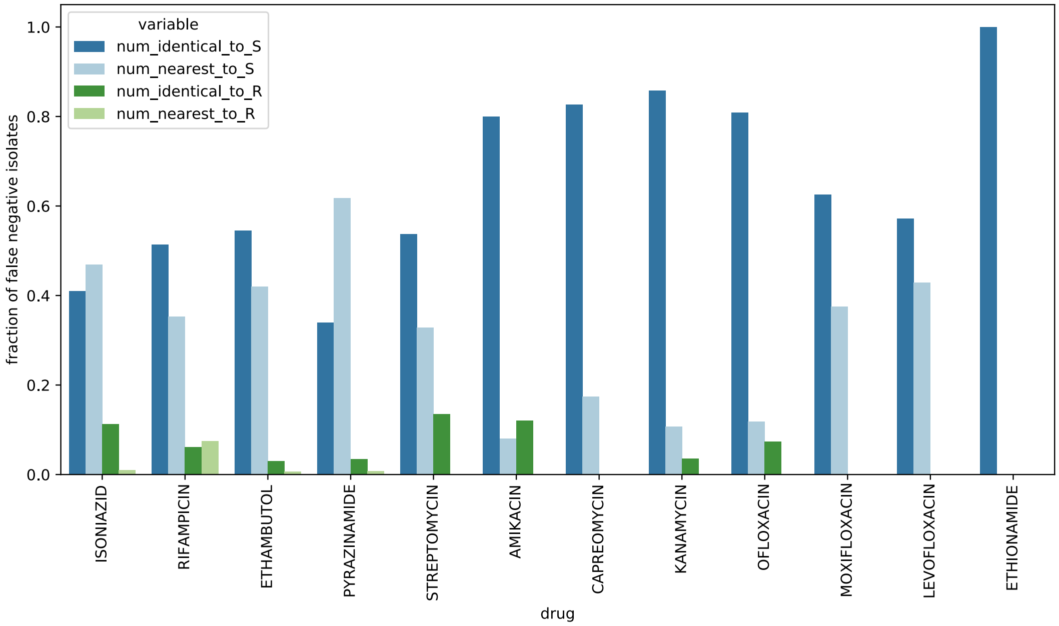

Supplementary Figure 4: Rifampicin saliency score plot for SD-CNN model. Plot of maximum DeepLIFT saliency scores across the rpoB gene (note that both *rpoB* and *rpoC* were included as inputs, only *rpoB* is shown here for resolution). The five highest saliency scores not previously known to cause resistance are highlighted in purple and visualized on the rpoB protein structure (PDB ID 5UH5) along with all higher-scoring known variants.

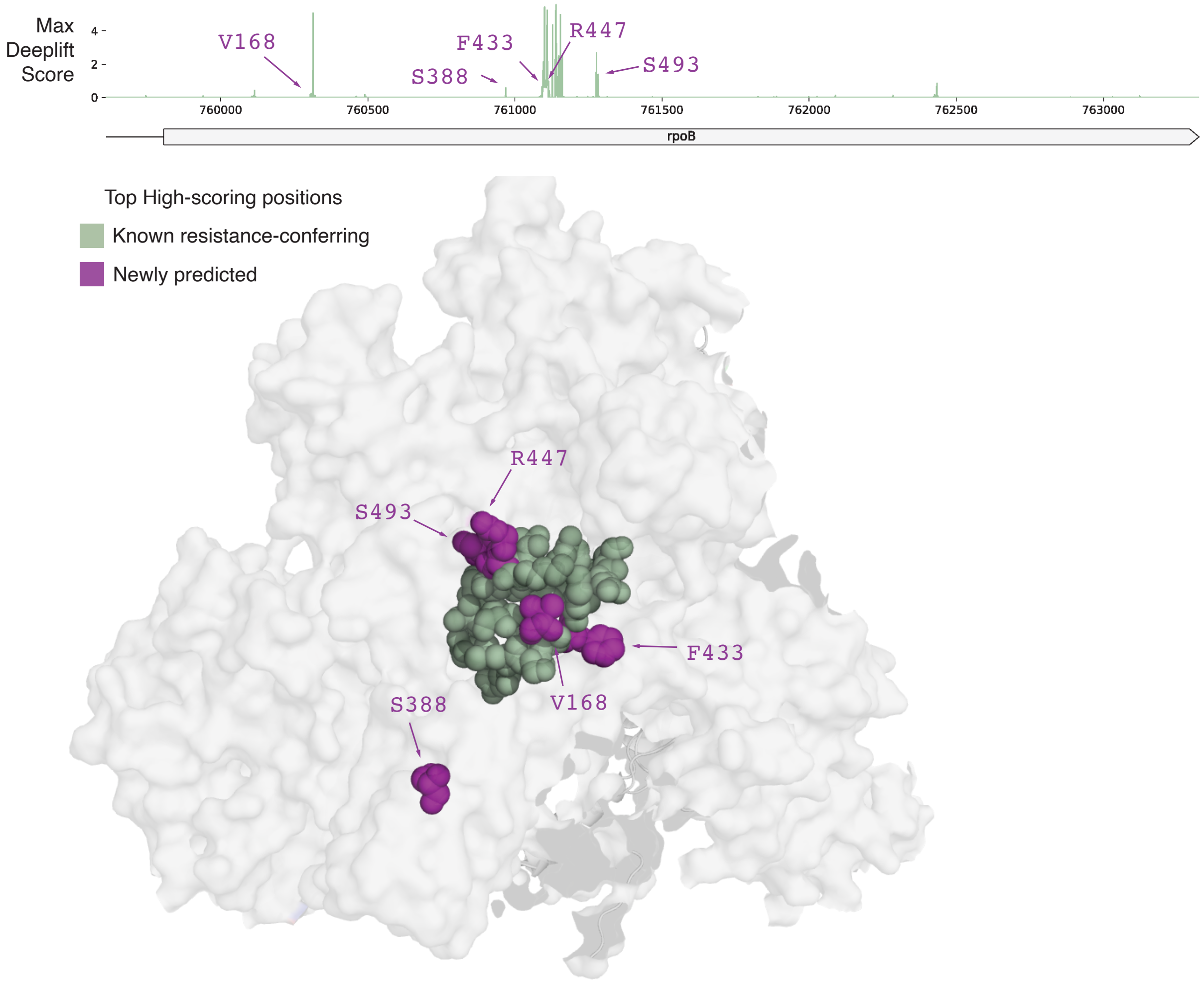

Supplementary Figure 5: MD-CNN validation accuracy and loss during cross-validation. Five-fold cross validation was performed on the MD-CNN to select the optimal number of epochs for training. The mean validation accuracy and validation loss are computed across the five splits. Final number of epochs selected is 150.

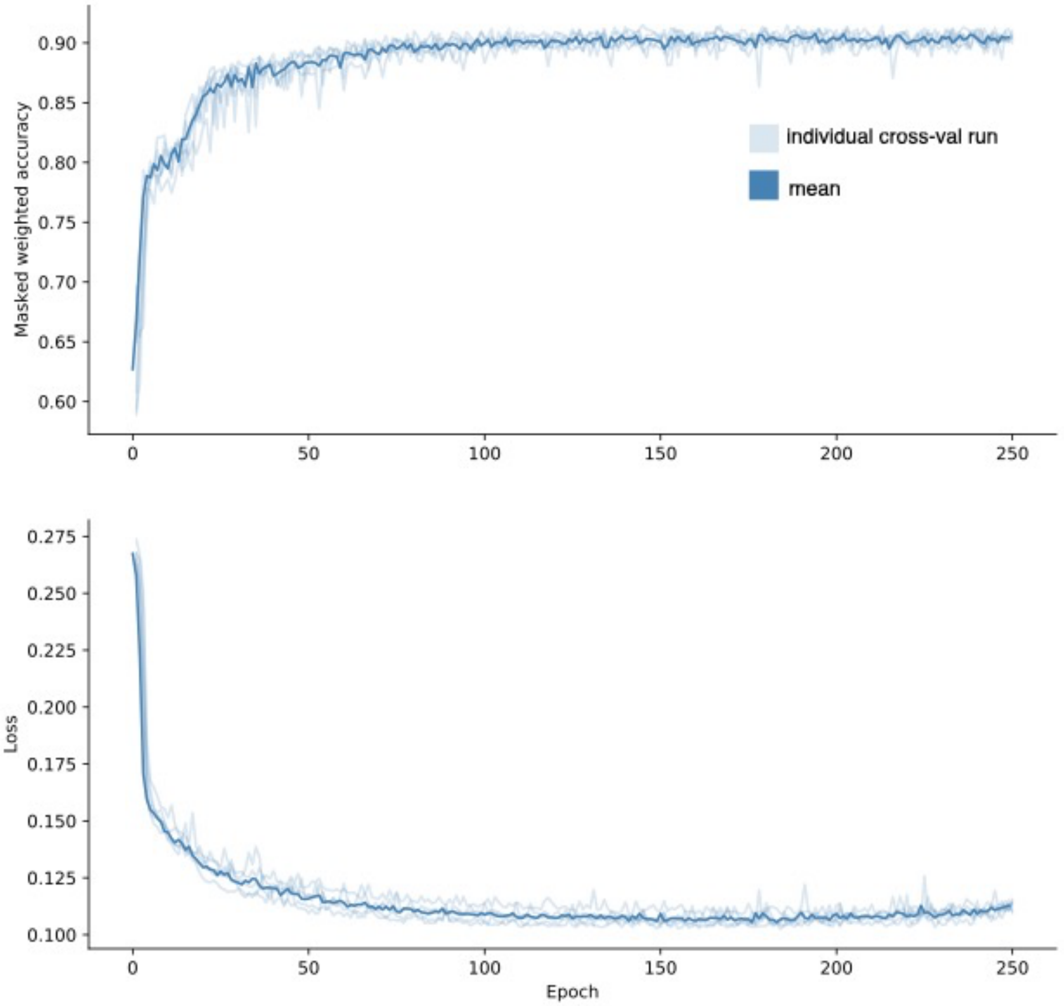

Supplement: Supplementary file 1 — Supplementary Information [file 41467_2022_31236_MOESM1_ESM.pdf]
