# Supplementary material for: A convolutional neural network highlights mutations relevant to antimicrobial resistance in Mycobacterium tuberculosis
Source: Nat Commun. 2022 Jul 2;13:3817. doi: 10.1038/s41467-022-31236-0 (PMC9250494; doi:10.1038/s41467-022-31236-0)
Supplement: Supplementary file 2 — Reporting Summary [file 41467_2022_31236_MOESM2_ESM.pdf]

nature portfolio

Corresponding author(s): Maha R. Farhat

Last updated by author(s): Jun 9, 2022

Reporting Summary

Nature Portfolio wishes to improve the reproducibility of the work that we publish. This form provides structure for consistency and transparency in reporting. For further information on Nature Portfolio policies, see our Editorial Policies and the Editorial Policy Checklist.

Statistics

For all statistical analyses, confirm that the following items are present in the figure legend, table legend, main text, or Methods section.

n/a	Confirmed	
☐	☒	The exact sample size (*n*) for each experimental group/condition, given as a discrete number and unit of measurement
☒	☐	A statement on whether measurements were taken from distinct samples or whether the same sample was measured repeatedly
☐	☒	The statistical test(s) used AND whether they are one- or two-sided<br>*Only common tests should be described solely by name; describe more complex techniques in the Methods section.*
☒	☐	A description of all covariates tested
☐	☒	A description of any assumptions or corrections, such as tests of normality and adjustment for multiple comparisons
☒	☐	A full description of the statistical parameters including central tendency (e.g. means) or other basic estimates (e.g. regression coefficient) AND variation (e.g. standard deviation) or associated estimates of uncertainty (e.g. confidence intervals)
☐	☒	For null hypothesis testing, the test statistic (e.g. *F*, *t*, *r*) with confidence intervals, effect sizes, degrees of freedom and *P* value noted<br>*Give P values as exact values whenever suitable.*
☒	☐	For Bayesian analysis, information on the choice of priors and Markov chain Monte Carlo settings
☒	☐	For hierarchical and complex designs, identification of the appropriate level for tests and full reporting of outcomes
☒	☐	Estimates of effect sizes (e.g. Cohen's *d*, Pearson's *r*), indicating how they were calculated

Our web collection on statistics for biologists contains articles on many of the points above.

Software and code

Policy information about availability of computer code

Data collection	Preprocessed Mycobacterium tuberculosis isolates and resistance phenotype data were obtained from Freschi et al, 2021 (https://doi.org/10.1038/s41467-021-26248-1) and Groeschel et al, 2021 (https://doi.org/10.1186/s13073-021-00953-4). The procedures, software, and<br>original data sources used by these manuscripts are described in the methods section. The following software packages were used: Kraken v0.10.6, minimap2-2.24 , BWA-MEM v0.7.17, Picard v2.9.2, SPAdes v 3.15.4, MAFFT v. 7.490, and trimmomatic v. 0.40.
Data analysis	The following software were used for model development and statistical analysis: Python 3.7.9, TensorFlow 2.3.0, CUDA 10.1, DeepLIFT v. 0.6.12.0, numpy v1.18.5,<br>and Scikit-learn v.0.23.2. All custom code is found in our github repository, https://github.com/aggreen/MTB-CNN.

For manuscripts utilizing custom algorithms or software that are central to the research but not yet described in published literature, software must be made available to editors and reviewers. We strongly encourage code deposition in a community repository (e.g. GitHub). See the Nature Portfolio guidelines for submitting code & software for further information.

Data

Policy information about availability of data

All manuscripts must include a data availability statement. This statement should provide the following information, where applicable:

- Accession codes, unique identifiers, or web links for publicly available datasets
- A description of any restrictions on data availability
- For clinical datasets or third party data, please ensure that the statement adheres to our policy

All code, processed input data, and saved model files are available on github, https://github.com/aggreen/MTB-CNN: v1.0. The processed strain phenotype data used in this study are available in MTB-CNN/input_data/master_table_resistance.csv and MTB-CNN/input_data/cryptic_phenotype_data.csv. The raw read data are publicly available for download from the NCBI using accession codes found in the processed strain phenotype data files. The processed FASTA files used as input to the CNNs are available in MTB-CNN/input_data/fasta_files and MTB-CNN/input_data/cryptic. The in silico mutagenized strains are available in MTB-CNN/input_data/dummy_strain_fasta_files. The trained MD-CNN and SDCNN models are available in MTB-CNN/saved_models. The model evaluation statistics generated in this study are provided in Supplementary Tables 1-4. The saliency score data generated in this study are provided in Supplementary Data 2 (MD-CNN) and Supplementary Data 3 (SD-CNN). Summaries and analysis of the saliency score data generated in this study are available in Supplementary tables 5-7.

Human research participants

Policy information about studies involving human research participants and Sex and Gender in Research.

Reporting on sex and gender	NA - no human subjects were used in the study
Population characteristics	NA
Recruitment	NA
Ethics oversight	NA

Note that full information on the approval of the study protocol must also be provided in the manuscript.

Field-specific reporting

Please select the one below that is the best fit for your research. If you are not sure, read the appropriate sections before making your selection.

☒ Life sciences ☐ Behavioural & social sciences ☐ Ecological, evolutionary & environmental sciences

For a reference copy of the document with all sections, see nature.com/documents/nr-reporting-summary-flat.pdf

Life sciences study design

All studies must disclose on these points even when the disclosure is negative.

Sample size	Sample size calculation was not performed. Sample size was determined by the number of publicly available Mycobacterium tuberculosis isolates with genotype and binary resistance phenotype data available at the initiation of the study.
Data exclusions	Our study used datasets of M. tuberculosis isolates that had undergone previous quality control for phenotype and genotype data. Therefore, no additional exclusions were performed.
Replication	Five-fold cross-validation was used during the training of the MD-CNN, SD-CNN, WDNN, and LogReg+L2 models to determine optimal hyperparameters. Generalizability of the MD-CNN and SD-CNN models was assessed by application to a held-out test dataset as described in the Methods and Results sections.
Randomization	Allocation of isolates from Freschi et al, 2021 into training or test dataset was performed randomly using numpy v1.18.5. Isolates from Groeschel et al, 2021 that<br>were not present in Freschi et al 2021 were allocated to the held-out test dataset, as these became available after the study was underway.
Blinding	It was not possible to blind researchers to whether specific strains were in the training or held-out test dataset as this would render model training impossible.

Reporting for specific materials, systems and methods

We require information from authors about some types of materials, experimental systems and methods used in many studies. Here, indicate whether each material, system or method listed is relevant to your study. If you are not sure if a list item applies to your research, read the appropriate section before selecting a response.

Materials & experimental systems

n/a	Involved in the study
☒	☐ Antibodies
☒	☐ Eukaryotic cell lines
☒	☐ Palaeontology and archaeology
☒	☐ Animals and other organisms
☒	☐ Clinical data
☒	☐ Dual use research of concern

Methods

n/a	Involved in the study
☒	☐ ChIP-seq
☒	☐ Flow cytometry
☒	☐ MRI-based neuroimaging
