# Supplementary material for: A convolutional neural network highlights mutations relevant to antimicrobial resistance in Mycobacterium tuberculosis
Source: Nat Commun. 2022 Jul 2;13:3817. doi: 10.1038/s41467-022-31236-0 (PMC9250494; doi:10.1038/s41467-022-31236-0)
Supplement: Supplementary file 3 — Description of Additional Supplementary Files [file 41467_2022_31236_MOESM3_ESM.pdf]

Title: Supplementary Data 1:
Description: results of cross-validation experiment on training data for each of four machine learning models on each of 13 drugs.

Title: Supplementary Data 2:
Description: top 1% of highest saliency positions for each drug detected by the MD-CNN model

Title: Supplementary Data 3:
Description: top 1% of highest saliency positions for each drug detected by the SD-CNN model
